# Single photon emitter deterministically coupled to a topological corner state

**DOI:** 10.1038/s41377-024-01377-6

**Published:** 2024-01-17

**Authors:** Mujie Rao, Fulong Shi, Zhixuan Rao, Jiawei Yang, Changkun Song, Xiaodong Chen, Jianwen Dong, Ying Yu, Siyuan Yu

**Affiliations:** 1 State Key Laboratory of Optoelectronic Materials and Technologies, School of Electronics and Information Technology, School of Physics, Sun Yat-Sen University, Guangzhou, 510006 China; 2 Hefei National Laboratory, Hefei, 230088 China

**Keywords:** Photonic crystals, Single photons and quantum effects

## Abstract

Incorporating topological physics into the realm of quantum photonics holds the promise of developing quantum light emitters with inherent topological robustness and immunity to backscattering. Nonetheless, the deterministic interaction of quantum emitters with topologically nontrivial resonances remains largely unexplored. Here we present a single photon emitter that utilizes a single semiconductor quantum dot, deterministically coupled to a second-order topological corner state in a photonic crystal cavity. By investigating the Purcell enhancement of both single photon count and emission rate within this topological cavity, we achieve an experimental Purcell factor of F_p_ = 3.7. Furthermore, we demonstrate the on-demand emission of polarized single photons, with a second-order autocorrelation function g^(2)^(0) as low as 0.024 ± 0.103. Our approach facilitates the customization of light-matter interactions in topologically nontrivial environments, thereby offering promising applications in the field of quantum photonics.

## Abstract (web summary)

We achieve the first deterministic coupling of a topological corner state with a single quantum dot, observing Purcell enhancement and polarized single-photon emission. This extends the effect of higher-order topological phase into the quantum realm.

## Graphical abstract

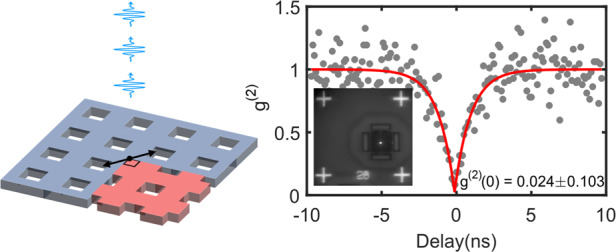

## Introduction

The radiation characteristics of a quantum emitter, including lifetime, intensity, and polarization, can be manipulated by modifying the surrounding photon environment. A widely adopted approach is through the implementation of cavity quantum electrodynamics (cQED), wherein a single quantum emitter is integrated into photonic microcavities boasting high-quality (Q) factors or small mode volumes^1,2^. These configurations have yielded significant advancements in the application of quantum information processing. In the linear weak coupling regime, they have led to notable enhancements of spontaneous emission^3–7^, resulting in high-quality quantum light sources. In the nonlinear strong coupling regime, fascinating effects like photon blockade^8^ and vacuum-induced transparency^9^ have been realized, providing a foundation for ultrafast qubit gate operations.

In the solid state, semiconductor quantum dots (QDs) deterministically coupled to optical microcavities, such as micropillar^10,11^, open-microcavity^5,12^, photonic crystal^13^, or circular Bragg grating^6,14^, have emerged as a highly promising platform for generating on-demand quantum states of light simultaneously with high efficiency, high purity and high indistinguishability. To gauge the strength of the coupling between a single QD and the cavity in cQED, an essential parameter of Purcell factor (F_p_) is employed. One of the significant advantages of the single-QD-in-microcavity structures lies in their compatibility with modern semiconductor fabrication processes, which allowing the creation of numerous devices with compact footprints on a single semiconductor chip. However, inherent challenges arise due to structural disorder or defects introduced during the fabrication process, which negatively affects the performance when coupled to single QDs^15^. In this context, topological optics present a promising solution due to their inherent topological robustness. For examples, the topological edge states present opportunities for customizing QD emissions, including topological slow light modes^16–19^ and chiral quantum interfaces^20–23^. While higher-order topological corner states in photonic systems have been implemented in different platforms, such as photonic crystals^24–30^, coupled ring resonators^31^, waveguide arrays^32^(typically used for transmission). The commonly used lattices for constructing corner states include a square lattice^24–29^, Kagome lattice^33–35,30^ and honeycomb lattice^36,37^. Compared to topological edge states, the higher-order topological corner state offers a smaller mode volume^26–29^, resulting a higher Purcell factor, or vacuum Rabi splitting even with a modest Q factor^38^. Among the corner states realized in different platforms, the corner states in photonic crystals have smaller mode volume and higher Purcell factor. Comparing to corner state in other lattices, corner states in square lattice with 2D SSH model have been proved that the emission can be tailored into waveguides for on-chip transmission^28^. Hence, square lattice 2D SSH photonic crystal supporting corner states become an excellent platform for quantum emitters.

However, challenges persist in coupling single QDs to highly confined topological cavities, primarily due to the random spatial distribution of QDs during their growth process. Previous attempts to implement such configurations encountered difficulties in achieving significant enhancement of light-matter interactions, resulting in Purcell factors below 1.8^27,39^. Thus, the deterministic interaction between a single QD and a topological cavity is highly desirable to fully exploit the advantageous properties of topology.

Here we take a step further by demonstrating the deterministic coupling of a single QD to a topological cavity featuring a zero-dimensional (0D) topological second-order corner state. By achieving resonance, we observed a notable Purcell enhancement both in lifetime measurement and in photoluminescence (PL) intensity. Through second-order correction measurements, we confirmed the anti-bunching properties of the emitted photons. Furthermore, the linear polarized cavity mode effectively redistributes the energy from charged exciton in the QD, resulting in a high degree of polarization. This finding opens up new possibilities for the realization of topological-cavity-based single-photon sources, which holds great potential for advancing topological quantum optics interfaces and exploring light-matter interactions at the single-photon level.

## Results

### Design of single QD in topological cavity

Our topological cavity is constructed based on 0D corner state, which emerges in a slab-type second-order topological photonic crystal (PhC) structure^24–26,40,41^. The band topology arises from the quantized edge dipolar polarization, which is characterized with a 2D Zak phase of $Z=({Z}_{x},{Z}_{y})$ that defined as:

$${Z}_{j}=\int d{k}_{x}d{k}_{y}{Tr}\left[{\hat{A}}_{j}({k}_{x},{k}_{y})\right]$$

**Fig. 1:**
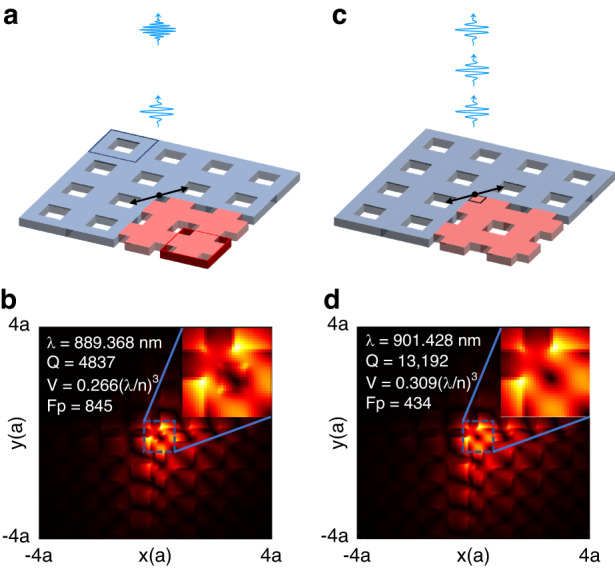
The theoretical scheme of the QD-in-topological cavity structure. (**a**) A schematic of the topological photonic crystal cavity with a quantum dot (QD), where a topologically nontrivial (red) PhC is surrounded by a trivial (blue) PhC. The black arrow represents a diagonally-polarized electric dipole source, representing a single QD; (**b**) The electric mode profile of the corner state at 889.368 nm, showcasing a Q factor of 4837, a mode volume of 0$.266{(\lambda /{n}_{{\rm{G}}{\rm{a}}{\rm{As}}})}^{3}$, and a Purcell factor of 845; (**c**) A schematic of the QD-topological photonic crystal cavity system without the center hole; (**d**) The electric mode profile of corner state without the center hole at 901.428 nm, showing a Q factor of 13192, a mode volume of 0$.309{(\lambda /{n}_{{\rm{G}}{\rm{a}}{\rm{As}}})}^{3}$, and a Purcell factor of 434

Here, *j* represents either the *x* or *y* direction, and ${\hat{A}}_{j}({k}_{x},{k}_{y})$ is the Berry connection^42^. Notably, the PhC structure features a distinct unit cell definition with a square lattice, as depicted by the red and blue regions in Fig. 1a. As a result, the corresponding Zak phases of ${(Z}_{x}{,Z}_{y})$ are (π, π) and (0,0), signifying the topologically nontrivial and trivial phase, respectively. Since the two different unit cells will coincide when the center of one of the unit cells is shifting by half a period for both *x* and *y* directions, they share the same band structure. Detailed structural parameters and the band structure are illustrated in Supplementary Information Fig. S1.

By combining the trivial (blue) and topologically nontrivial (red) PhCs together, as illustrated in Fig. 1a, a corner state deterministically emerges as a convergence of the two sets of 1D interface polarization. Figure 1b displays the calculated electric field profile of the corner state, which is excited by a diagonally polarized electric dipole source (black arrow, also considered as single QD) at the resonant wavelength of 889.368 nm. It is found that the corner state is tightly localized, with a calculated Q factor of 4837, a small mode volume of $0.266{(\lambda /{n}_{{\rm{G}}{\rm{a}}{\rm{As}}})}^{3}$, in which ${n}_{{\rm{G}}{\rm{a}}{\rm{As}}}=3.41$ and thus a Purcell factor of 845.

However, in such cavity, the single QD is situated very close to the dry-etched surface, which may result in spectral diffusion or blinking due to coupling with surface states and charge traps^15^. To address this issue, we leverage the robustness of the corner state and modify the design by removing the center airhole, as depicted in Fig. 1c. As the corner state is inherently guaranteed by the topological property of the edge dipolar polarization, it remains unaffected by weak perturbations, such as the removed airhole^29^. Figure 1d illustrates the corner state profile with the center hole fulfilled. The corner state remains almost intact, albeit with a slight shift in the resonant wavelength to 901.428 nm. Notably, the Q factor of the corner state cavity significantly increases to 13192, while the mode volume expands to $0.309{(\lambda /{n}_{{\rm{G}}{\rm{a}}{\rm{As}}})}^{3}$. However, due to the reduced overlap between the QD and the cavity mode when the center hole is filled, the Purcell factor decreases to 434. Nevertheless, the design without center hole offers several advantages, including a higher Q factor, a modest mode volume and a larger distance (~100 nm) between the QD and the etched surface.

### Fabrication and characterization of the topological cavity

**Fig. 2:**
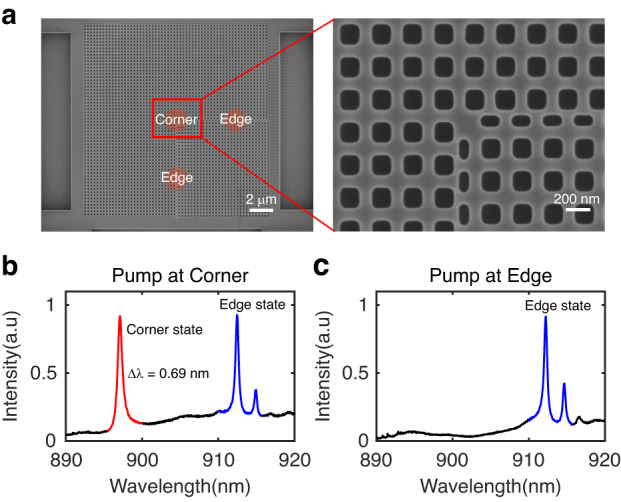
The fabricated sample and the performance of the topological cavity. **a** The scanning electron microscopy (SEM) images of a topological device patterned on a GaAs wafer. The inset provides a magnified view of the red area, highlighting the cavity’s location hosting the corner state. **b** The photoluminescence image captured under high pump power at the corner region. The red area indicates the presence of the cavity mode associated with the corner state, while the blue area corresponds to the modes associated with the 1D edge states. **c** The photoluminescence image captured under high pump power at the edge region. The peak of corner state vanishes while the edge states remain present

The devices are fabricated on a 200-nm-thick GaAs slab containing a monolayer of self-assembled InAs QDs grown by molecular beam epitaxy. Beneath the membrane, there exists an 1800 nm thick sacrificial layer of Al_0.8_Ga_0.2_As layer on the GaAs substrate. The pattern mask is created using electron beam lithography and then transferred to the GaAs membrane using inductively coupled plasma etching. To achieve the free-standing structure, the sacrificial layer is removed via hydrofluoric acid wet etching. A cleaning procedure involving hydrogen peroxide followed by a potassium hydroxide solution is carried out to ensure the thorough removal of residues from both wet and dry etching processes^43^ (see Method for details). Figure 2a displays a representative top-view scanning electron microscope (SEM) image of the fabricated topological corner state cavity, containing 40×40 PhC unit cells. The inset in the figure provides an enlarged view of the corner region, where a corner state resides at the interface between the topological nontrivial and trivial PhCs.

To characterize the cavity modes, we initially fabricate the device in the high-density region of QDs. Micro-photoluminescence (μ-PL) measurements were conducted using a 785-nm continuous-wave (CW) laser at 4.2 K (see Supplementary Information Fig. S2 for details). Figure 2b displayed a high-power μ-PL spectrum of the device, excited and collected from the corner region. The sharp peak observed at 898 nm corresponds to the resonant wavelength of corner state, which is in good agreement with the simulation result presented in Fig. 1d. Concurrently, the peaks in the range of 910-920 nm are originated from the 1D edge states. Our simulation results confirm that the edge states are located on the redshifted wavelength side of the corner state, consistent with experimental findings (see Supplementary Information Fig. S4 for details). Notably, as the excitation and collection points shift towards the edge region, the corner state ceases to exist. Nevertheless, the peak of edge states, induced by Fabry-Perot resonances, remains evident, as illustrated in Fig. 2c. As excitation and collection move away from the corner and into the bulk region, both peaks vanish completely. Moreover, the linewidth of the corner state is determined to be 0.69 nm, with a spectral resolution of 0.02 nm, corresponding to an experimental Q value of 1300. Thus, our topological corner state microcavities have been successfully fabricated and can be effectively integrated with a single quantum dot in subsequent steps.

### Single QD deterministically coupling to a corner state

**Fig. 3:**
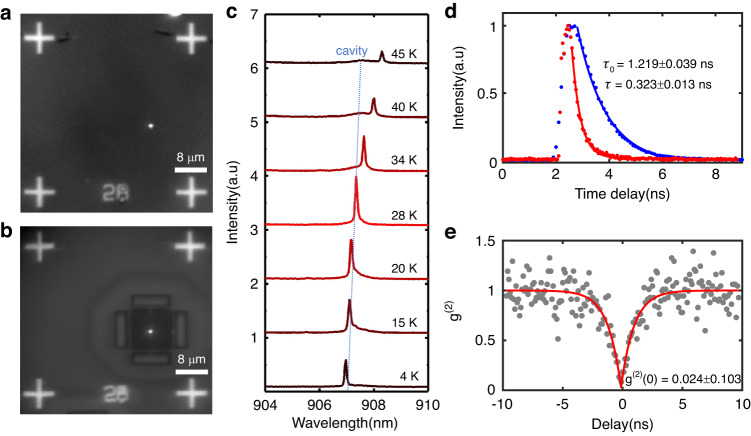
Single QD positioning and coupling to corner state. **a**, **b** Fluorescence images captured before and after the fabrication of the topological cavity, revealing the precise positioning of a single QD in the vicinity of the corner state. **c** The PL spectra of the QD as a function of temperature, measured across the corner state cavity. At 28 K, the QD is in resonance with the cavity. **d** Decay curves comparing the QD on resonance with the cavity (red) and the QD in bulk GaAs material (blue), revealing a Purcell factor of 3.7. **e** An intensity-correlation histogram obtained using a Hanbury Brown and Twiss setup, under above-band continuous wave (CW) excitation. The histogram demonstrates the antibunching behavior of the emitted single photons

Effectively coupling a single quantum dot (QD) with the corner state requires critical spectral and spatial alignment of the QD emission with the cavity mode. However, addressing this issue has been challenging in previous reports due to the nanoscale geometry of the corner state cavity. In our study, we overcome this challenge by leveraging our novel cavity design and wide-field PL imaging technique. This enables us to spatially overlap individual QDs with the cavities. The position of pre-selected single QDs is precisely determined relative to alignment marks, with an uncertainty of approximately 10 nm^44–46^. Subsequently, we deterministically fabricate a topological cavity around the target QD. Figures 3a, b display the fluorescence image of our device before and after the cavity fabrication, where a targeted single QD (bright spot) is clearly visible at the center of the fabricated corner state cavity.

To demonstrate the light-matter interaction in a topological cavity, we successfully coupled the exciton line from a single QD to a corner state under Purcell regime. By adjusting the temperature, we were effectively tuned the target exciton across the resonance of the corner state, as illustrate in Fig. 3c. At 4 K, the wavelength of the exciton is measured to be 907.0 nm, while the resonance of the corner state is observed at 907.8 nm under high pumping power. The cavity exhibits a full width at half maximum (FWHM) of 0.54 nm, corresponding to a Q factor of 1681 (Supplementary Information Fig. S3). As temperature increases, the coupling strength between the exciton and corner state also escalated, given their gradual approach towards each other. The mode of corner state redshifts as temperature increases but at a slower rate than the exciton line. When reached resonance at 28 K, a maximum enhancement of PL intensity by a factor of 3.2 is observed. However, with further temperature increase, radiation suppression occurred, resulting in a subsequent decrease in PL intensity.

To comprehensively investigate the Purcell enhancement, we performed time-resolved measurements using a fast single-photon avalanche diode (SPAD) with an instrument response of 35 ps. A pulsed laser operating at a wavelength of 700 nm with a repetition frequency of 81 MHz was utilized. Figure 3d presents a comparison of the lifetimes between the QD on resonance and in bulk GaAs. Notably, on resonance, the lifetime is significantly reduced to ~323 ps (red curve). In contrast, the average lifetime for QDs in the slab is ~1.219 ns (blue curve). As a result, we achieved a Purcell factor of about 3.7. This observation convincingly demonstrates the exceptional enhancement effect exerted by the topological corner state on single QD. However, the experimental Purcell factor deviates significantly from the simulated values due to three primary reasons. Firstly, fabrication imperfections result in a Q factor approximately an order of magnitude lower than in the simulations. Secondly, the relatively small mode volume implies that even a slight positional deviation can lead to QDs not being optimally positioned within the mode field. Finally, the slow relaxation process stemming from above-band excitation further diminishes the Purcell factor^3^.

To verify the emission of single photons, we performed a Hanbury Brown and Twiss (HBT) correlation measurement under above-band excitation, using a CW laser with a wavelength of 785 nm. The collected photons were directed to a fiber-based beam splitter and APDs via single mode fiber. Figure 3e presents a second-order autocorrelation data recorded by a time correlator, indicating a low multi-photon probability of g^(2)^(0) ~ 0.024 ± 0.103. This result confirms the anti-bunching behavior of the emitted single photons.

### Characterization of polarization of emitted photons

**Fig. 4:**
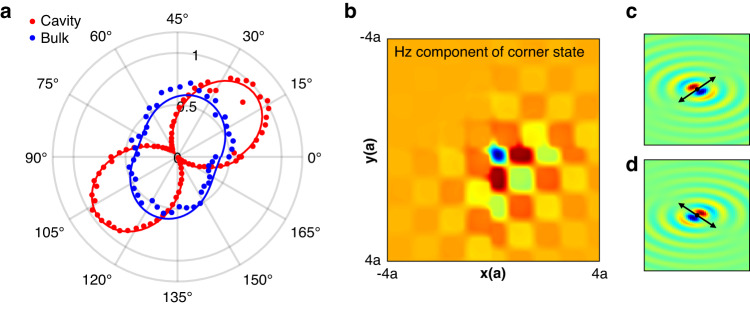
Corner state cavity symmetry and polarization characterization. **a** The intensity of emitted photons from the charged exciton X^+^ plotted as a function of the angle of the half-wave plate, showing both on-resonance with the cavity mode (red) and in bulk material (blue). The linear polarizabilities are calculated as 96% and 61%, respectively. **b** The H_z_ component of the corner state mode profile, which exhibits a specific pattern and symmetry, which providing crucial insights into the polarization effects of the cavity. **c**, **d** H_z_ component of dipole radiation for two different orientations of the dipole: 45^o^ (**c**) and 135^o^ (**d**). The direction of the arrow indicates the orientation of the dipole. By comparing these plots with the H_z_ component of the corner state mode profile (shown in Fig. 4b), the coupling and modulation effects of the cavity on the dipole with different orientations can be analyzed

To investigate the diverse coupling effects between the corner state and dipole with different polarizations, we utilized a positively charged exciton X^+^, which is circularly polarized. The emitted photons were passed through a half-wave plate followed by a linear polarization plate. As depicted in Fig. 4a, when the exciton line resonates with the corner state, we observed variations in PL intensity as the angle of the half-wave plate changed, yielding a measured linear polarizability of 96%. For comparison, we also measured the variation in the PL intensity of the charged exciton in the bulk material (depicted as blue dots in Fig. 4a). The measured linear polarizability decreases to 61%, indicating its circular polarization nature. The slight elliptical polarizability observed can be attributed to the inherent asymmetry of the QD. These observations clearly demonstrate the occurrence of the coupling modulation effect between the corner state cavity and dipoles with different orientations, which is advantageous for achieving polarized single-photon emission. In Fig. 4b, we present FDTD simulation results depicting H_z_ component of corner state exhibiting even symmetry along 135^o^. This symmetry aligns parallel to that of a dipole oriented at an angle of 45 ^o^ (Fig. 4c), while perpendicular to that of a dipole oriented at an angle of 135^o^ (Fig. 4d). Consequently, the cavity enhances the emission from the 45^o^-oriented dipole, while simultaneously suppressing that from the 135^o^-oriented dipole. Our simulations support the experimental observations: employing a dipole angled at 45^o^ yields Purcell factor value reaching up to 434, resulting in radiation enhancement; however, using a dipole angled at 135^o^ only leads to Purcell factor value as low as 0.1, causing radiation suppression (Supplementary Information Fig. S5).

## Discussion

In conclusion, we have demonstrated the deterministic fabrication of corner state cavities around target QDs using a precise positioning technique. By employing temperature tuning, we have achieved an impressive on-resonance Purcell factor of 3.7. The device also exhibits a single photon purity g^(2)^(0) as low as 0.024 ± 0.103, indicating the anti-bunching characteristic of the emitted photons. Furthermore, we verified the efficient dipole polarization modulation capability of the corner state cavity. In the classical domain, lasing action of topological corner states has been observed^47–49^. This discovery expands the potential of them for advanced applications in manipulating light-matter interactions at the quantum level.

Moving forward, further increasing the coupling strength can lead to the transition from Purcell enhancement to vacuum Rabi splitting (See details in Supplementary Information Fig. S6). Enhancing the quality factor of the corner state cavity by atomic layer deposition (ALD)^15,29,50^ or further parameter optimization^29^ can contribute to increased Purcell enhancement (See details in Supplementary Information Fig. S7). Moreover, the inherent polarization selection property of the corner state cavity offers the potential to significantly enhance the convenience of resonance fluorescence (RF) measurements and even surpass the conventional limit of 50% orthogonal RF efficiency^4,5^. In addition, the topological corner states can be extended into other quantum emitters^38^^,^^51^. While more complex corner states^52^ can be utilized to enhance the excitation process^11^.

## Materials and methods

### Numerical simulation

The Q factor, mode volume, Purcell factor and mode profile were calculated by the Finite-Difference Time-Domain (FDTD) method. The 3D simulation was employed with 40 × 40 cell arrays. The refractive index of GaAs material is assumed to be 3.41. Perfectly matched layer domains are used to reduce the reflection from the simulation boundries. The simulation time is set to be 10^6^ fs to ensure full convergence of the simulation.

### Sample fabrication

To acquire the position of QDs by optical positioning technique, the mark arrays with 10 nm Ti and 100 nm Au are first created on the surface of the sample by the standard E-beam lithography, metal deposition, and lift-off processes. Next, the sample was spin-coated with a positive resist (AR-P 6200). The resist was then exposed to a 100 kV electron beam using a VISTEC EBPG5000 ES PLUS electron-beam lithography (EBL) system. After the develop process, the pattern is transferred to the sample by inductively coupled plasma with gases of SiCl_4_, Ar and N_2_. After removing the residual photoresist by oxygen plasma surface treatments, the sample is dipped into the 10% hydrofluoric acid for 20 s to remove the sacrificial layer. A cleaning procedure with 30% H_2_O_2_ for 60 s followed by 60 s water rinse and 120 s 20% KOH can remove the residues completely.

### Supplementary information

Supplemental Material
