# Supplementary material for: Single photon emitter deterministically coupled to a topological corner state
Source: Light Sci Appl. 2024 Jan 17;13:19. doi: 10.1038/s41377-024-01377-6 (PMC10792022; doi:10.1038/s41377-024-01377-6)
Supplement: Supplementary file 1 — Supplemental Material [file 41377_2024_1377_MOESM1_ESM.pdf]

Supplementary Information for

Single photon emitter deterministically coupled to a topological corner state

Mujie Rao[1#], Fulong Shi[1#], Zhixuan Rao[1], Jiawei Yang[1], Changkun Song[1], Xiaodong Chen[1], Jianwen Dong[1]*, Ying Yu[1,2]*, Siyuan Yu[1,2]

[1] *State Key Laboratory of Optoelectronic Materials and Technologies, School of Electronics and Information Technology, School of Physics, Sun Yat-Sen University, Guangzhou 510006, China*

[2] *Hefei National Laboratory, Hefei 230088, China*

**Corresponding author*: dongjwen@ mail.sysu.edu.cn ; yuying26@mail.sysu.edu.cn

[#]These authors contributed equally to this work

Section 1 Band structure of 2D Photonic crystal (PhC) slab.

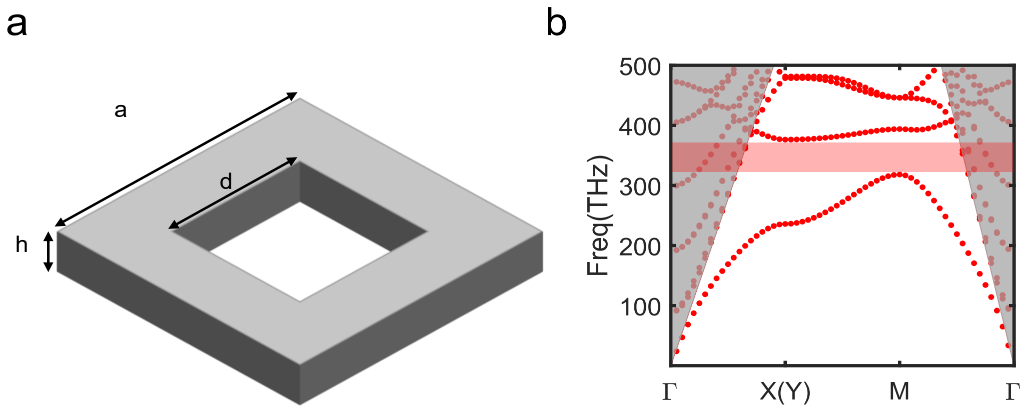

Figure S1 (a) An illustration of photonic crystal (PhC) unit cell, where 'a' represents the in-plane lattice constant, 'd' stands for the width of the square hole, and 'h' denotes the height of the slab; (b) The TE-like bulk band structure of the square freestanding photonic crystal slab, revealing a band gap spanning from 797.2 nm to 942.9 nm.

The schematic illustration of the photonic crystal (PhC) unit cell is presented in **Fig.S1a**, with a lattice constant of a = 270 nm, square hole width of d = 176 nm, and slab height of h = 200 nm. The transverse electric (TE) band diagram of a freestanding GaAs PhC slab (n=3.41) is presented in **Fig. S1b**. The diagram was calculated using the MIT Photonic Bands (MPB) software and the 3D plane wave expansion method[1]. The wavelength of photonic bandgap is adjusted to fall within the range of 797.2 nm to 942.9 nm, to cover the spectral range of the single InAs QDs.

Section 2 Optical setup.

The optical setup and sample characterization system are depicted in **Fig. S2**. The sample is placed on three-dimensional nanopositioners inside a cryostat operating at a base temperature of 4.2 K. The sample is pumped using either a 785 nm continuous-wave (CW) laser or a 700 nm pulsed laser with a repetition frequency of 81 MHz through a 50× objective with a numerical aperture (NA) of 0.65. The emitted photons are collected through the same objective and then filtered using a long-

pass filter. Subsequently, the collected photons pass through a half-wave plate and then a linear polarizer for polarization properties characterization. Finally, the photons are either directed to a spectrometer for spectral analysis or passed through a 1 nm band-pass filter. The filtered photons are then sent to an avalanche photodiode for lifetime characterization and Hanbury-Brown-Twiss (HBT) measurements.

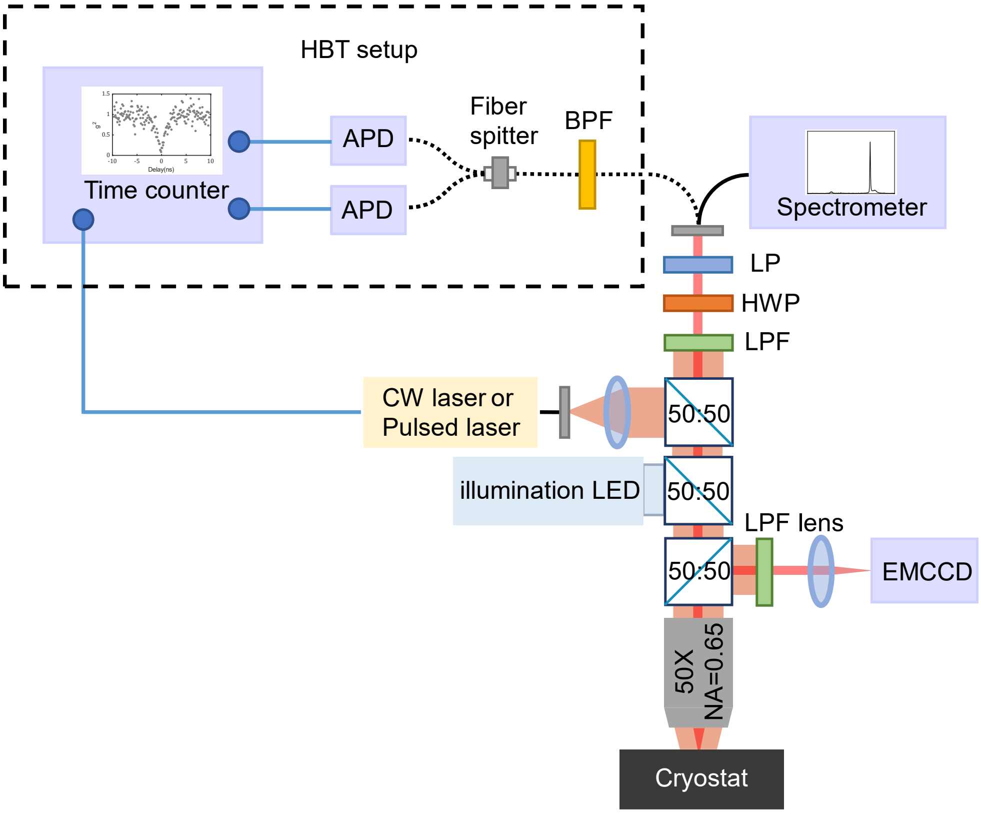

Figure S2 Optical setup. LPF: long pass filter. HWP: half-wave plate. LP: linear polarizer. BPF: band pass filter. APD: avalanche photo diode.

Section 3 Target exciton variation with temperature.

Fig. S3a displays the photoluminescence (PL) spectra of the QD under low-power pumping by a 785 nm CW laser. The target exciton is observed at a wavelength of 907.0 nm, while the corner state cavity is indicated by an orange arrow. The inset in **Fig. S3a** presents the spectrum of the corner state cavity under high pumping power. It reveals a peak position at 907.8 nm with a linewidth of 0.54 nm, corresponding to a quality factor of 1681. **Fig. S3b** illustrates the comprehensive variation of both the exciton wavelength and intensity with temperature in **Fig. S3a**. As the temperature increases, the wavelength of the target exciton gradually redshifts. At 28 K, the exciton is in resonance with the corner state, resulting in the highest intensity counts and a maximum enhancement of the PL intensity by a factor of 3.2. However, as the temperature further increases, the exciton line detunes from the corner state mode, leading to a gradual decrease in the intensity of the emitter.

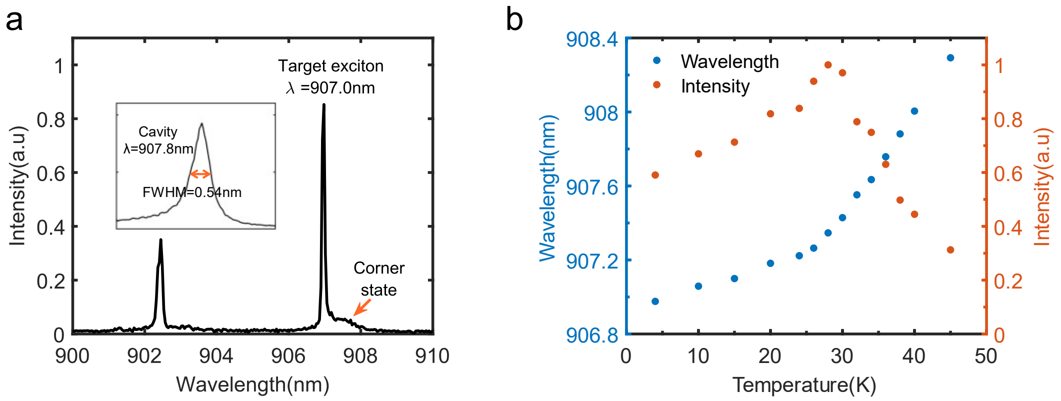

Figure S3 (a) PL spectra of the target exciton and the corner state mode. (b) PL intensity and wavelength of the exciton indicated in (a).

Section 4 Simulation of edge states.

We placed a 45°-oriented dipole source at the location of the corner and set the point time monitor at the same location to collect the spectrum. Besides the resonant peak of the corner state mode at 901.428 nm that we discussed in the text, we have also observed additional resonances at slightly redshifted wavelengths of 922.228 nm and 923.683 nm in the Finite Difference Time Domain (FDTD) simulation, as shown in Fig.S4a. These resonances exhibit a similar mode profile, as depicted in **Fig. S4b-c**. The downward resonance peak at 923.683 nm results from the spatial position of the point time monitor at the absorption of the edge state. We considered the presence of multiple resonances can be attributed to the finite number of periods in our sample, in which the boundary leads to reflections of the edge states, which is aligned with the previous reports[2,3]. These reflections give rise to Fabry-Perot resonances, which affect the quality factors (Q factors) of the observed resonances. Specifically, the resonance at 922.228 nm has a higher Q factor of 46084, while the resonance at 923.683 nm has a lower Q factor of 2833 in our case.

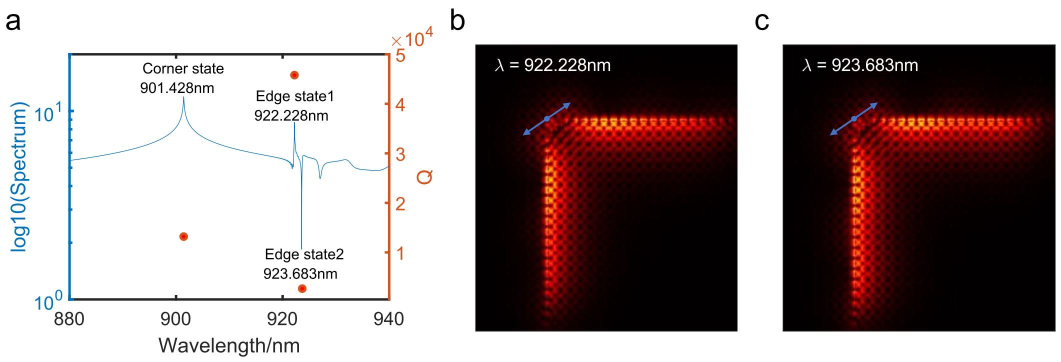

Figure S4 (a) The spectrum of the time monitor in FDTD simulation. The resonance lines include a corner state as well as two edge states. (b-c) The mode profiles on the resonance of edge state modes at the wavelengths of 922.228 nm and 923.683 nm. A 45°-oriented dipole source is applied, and the blue arrows indicate the position of the source and point time monitor.

Section 5 Simulation of different orientated dipoles

The corner state cavity has different modulation for different orientations of dipole. As shown in **Fig. S5**, when the dipole is orientated at 45°, FDTD simulation exhibits a Purcell factor of 434 and the radiation is enhanced. Conversely, when the dipole is oriented at 135 degrees, the Purcell factor is less than 1, leading to radiation suppression. As described in the main text, we attribute this behavior to the different overlap between the corner state mode and dipoles with distinct orientations.

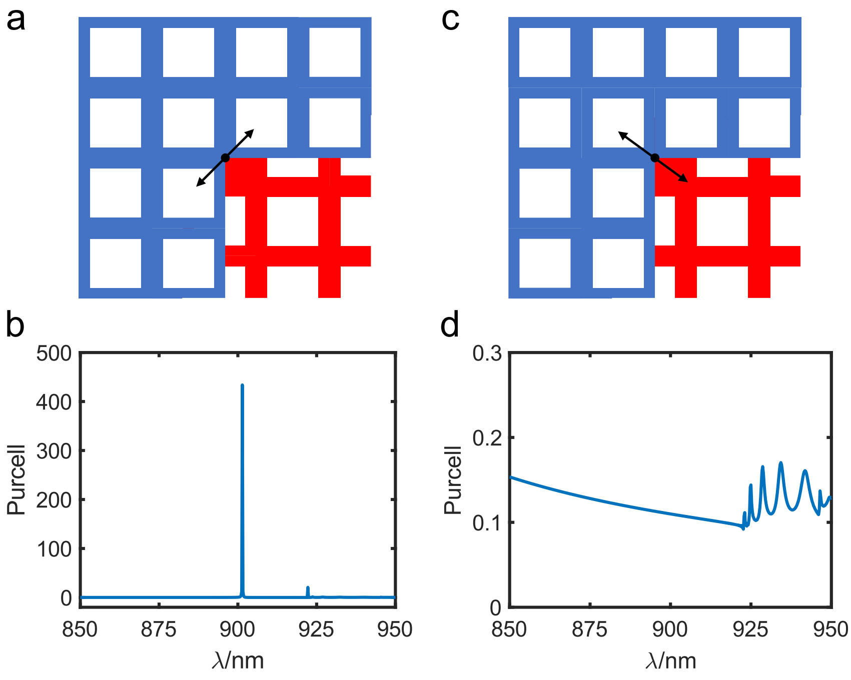

Figure S5 (a) Schematic of 45°-oriented dipole excite at the center. (b) The Purcell factor of dipole in (a), resulting in radiation enhancement. (c) Schematic of 135°-oriented dipole excite at the center. (d) The Purcell factor of dipole in (c), resulting in radiation suppression.

Section 6 Discussion on Strong Coupling

In our experiments, we did not observe Vacuum Rabi splitting, which indicates the coupling strength g between the emitter and photon is larger than the combined emitter decay rate γ_{dot} and the cavity field decay rate κ_{cavity}. However, given the high Q factor and small volume of the corner state cavity, we conducted simulations and experiments to analyze the potential of this microcavity for strong coupling.

In theory[4], we extract QD dipole moment μ via $1/\tau = n\omega^3\mu^2/3\pi\varepsilon_0\hbar c^3$, where $\tau \sim 1$ ns is the lifetime of QD in bulk GaAs material, $n \sim 3.41$ is the refractive index of GaAs material, ω is the transition angular frequency, ε_0 is the permittivity in free space, $\hbar$ is the reduced Planck constant and c is the speed of light in vacuum. According to FDTD simulation, the volume of corner state mode is $V = \int \varepsilon E^2 dV / max(\varepsilon E^2) = 0.00572\ \mathrm{um}^3 = 0.309(\lambda/n)^3$. Based on the aforementioned dipole moment and mode volume we can calculate the theoretical coupling strength $g = \mu(\hbar\omega/2\varepsilon_0)^{1/2}/n\hbar = 358$ GHz.

In the experiment, the corner state cavity mode is predominantly centered around 900 nm, exhibiting a half-height width of 0.6 nm, corresponding to a decay rate of $\kappa_{\mathrm{cavity}} = 222$ GHz. Due to $g \sim 1.61\kappa_{\mathrm{cavity}}$ and the decay rate of QD γ_{dot} is much smaller than κ_{cavity}, the coupling strength

exceeds the threshold for strong coupling, which is defined as: $g \geq (\kappa_{\text{cavity}} + \gamma_{\text{dot}})/4$. The theoretical spontaneous radiation spectra of QD is shown in **Fig. S6a**[5], revealing a prominent vacuum Rabi splitting phenomenon. In practice, due to the small size of the mode volume, it is challenging to ensure that the QD is precisely positioned at the crest of the mode. The Purcell factor is given by[6]:

$$Fp = \frac{3Q}{4\pi^2 V}\frac{|\mu \cdot E(r)|^2}{|\mu|^2|E_{max}|^2} = 3.7$$

Define $V^* = \frac{v|\mu|^2|E_{max}|^2}{|\mu \cdot E(r)|^2}$, thus we get an actual mode volume $V^* = 23.96\ \text{um}^3 = 1303(\lambda/n)^3$. Therefore, the actual coupling strength is $g_{\text{exp}} = 5.53\ \text{GHz}{\sim}0.025\kappa_{\text{cavity}}$. Thus, the conditions to achieve strong coupling regime have not yet been reached. **Figs. S6b-c** illustrate the real and imaginary parts of the theoretical spontaneous radiation transition from the Purcell enhancement to the Vacuum Rabi splitting, respectively[4,7]. The experimental values are indicated by red stars, while the theoretical values are represented by red circles. This observation suggests that the corner state cavities possess the potential to achieve strong coupling even at existing Q levels. Further precise positioning of the QD within the cavity mode remains a challenge that needs to be addressed to fully realize the strong coupling regime.

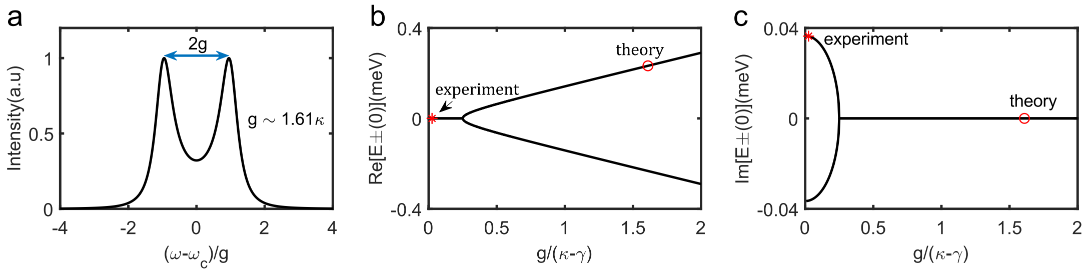

Figure S6 (a) The normalized emission spectra in theory when the QD is in resonance with the cavity. The spectra display a vacuum Rabi splitting phenomenon. (b-c) Illustration of the energy (b) and linewidth (c) transition process transition from Purcell enhancement to vacuum Rabi splitting.

Section 7 Optimization of the corner state

To further increase the Q factor of the corner state, thus improving the Purcell factor and enabling the observation of strong coupling phenomena, we conducted a series of parameter sweeps[3]. We performed parameter sweeps by three main methods: adjusting the width of the boundary row or column air holes, shifting the boundary row or column air holes, and varying the spacing between the two PhCs. **Figs. S7a-c** depict the schematic of the structure before and after the modification, with the dashed line indicating the original structure. **Figs. S7d-f** display the Q factor of the corner state and the resonance wavelength after the corresponding structural changes.

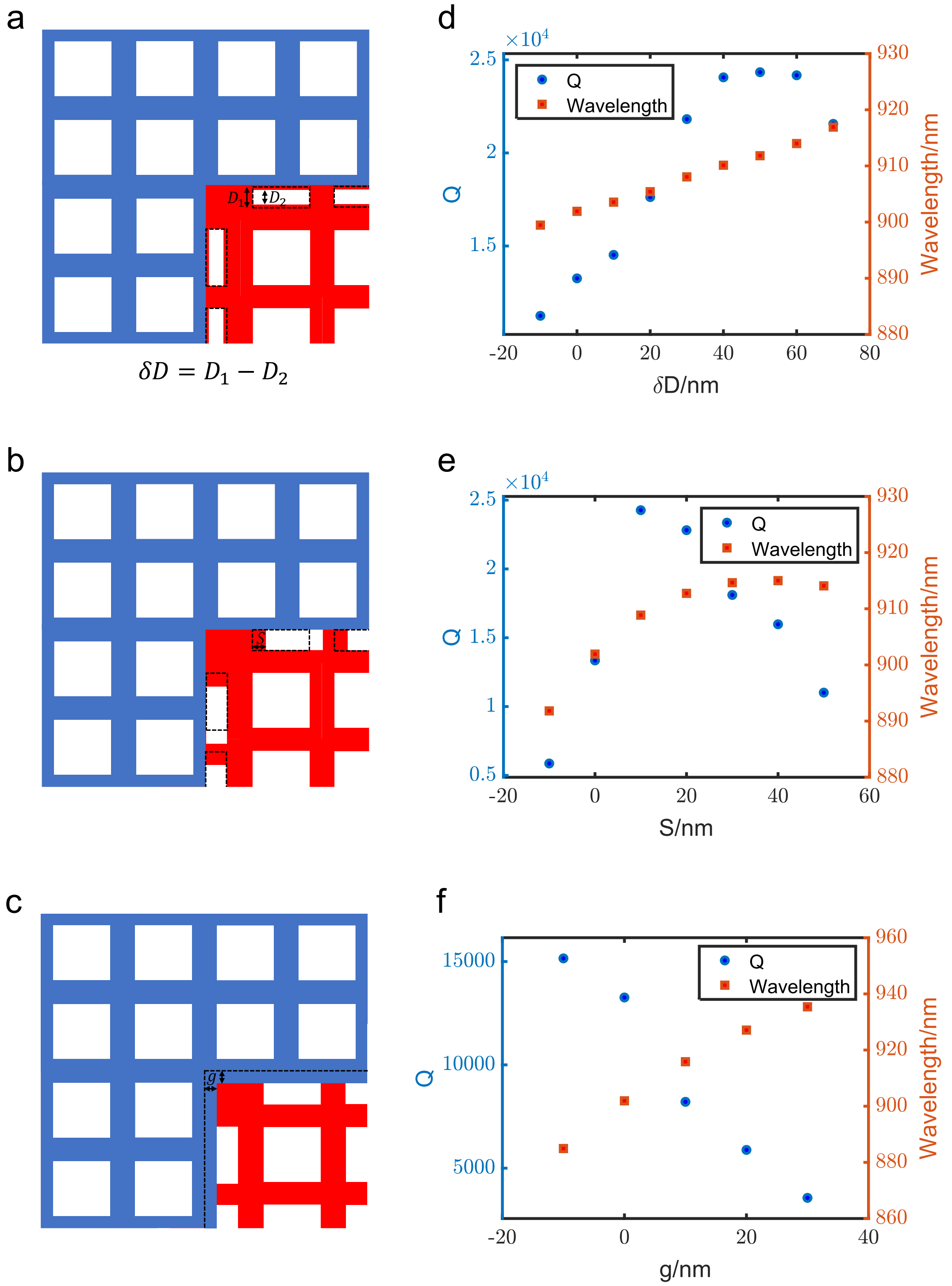

Figure S7 (a-c) The schematic of the corner state structure before and after the modification, with the dashed line representing the original structure. (a) Schematic of adjusting the width of the boundary air holes. (b) Schematic of shifting the boundary air holes. (c) Schematic of tuning the space between the two PhCs. (d-f) The simulated Q factor and resonance wavelength of the corner state are shown as a function of the modified parameters in (a-c).

Section 8 Robustness of the corner state

The robustness against disorder and defects is a key advantage of topological photonics. Our design capitalizes on the inherent robustness of the corner state by deliberately incorporating a

defect at the corner. To further investigate the impact of various defects on the wavelength and Q factor of the corner state, we conducted simulations including the introduction of bulk defects in both trivial and nontrivial lattices, as well as edge defects at the boundary between trivial and nontrivial lattices. The robustness of the corner state is demonstrated by analyzing the effects of bulk and edge defects in the original sample with the center airhole reserved, as depicted in Fig. S8.

The original Q factor of the corner state in the sample with the center airhole reserved is 4837, and the original wavelength is 889.368 nm. In Figure S8 (a-c), individual holes are removed to create defects. The bulk defect is introduced by removing the air hole along the diagonal. Owing to the structure's symmetry along the diagonal, the removal of the edge air hole in either the x-direction or the y-direction produces the same effect. Each removed hole is labeled as Mx, Nx, or Lx, with x representing the serial number based on the distance from the corner. The impact of defect location on the wavelength of corner states is consistent across the three types of defects. The closer the defect is to the corner, the greater the redshift in the corner state wavelength. The impact of bulk defects in the trivial lattice Mx on the Q factor is negligible, with the exception for M1. For both edge defects and bulk defects in the nontrivial lattice, the Q factor approaches the level without defects as the distance from the corner increases, except for L1 defects. Furthermore, an increase in the Q factor is observed after removing M1 and L1, potentially attributed to a smoother spatial variation near the corner state cavity. This observation is analogous to the increase in Q from 4837 to 13192 after removing the hole in the center. Despite defects closest to the corner, such as M1, N1, and L1, the corner state maintains its presence, demonstrating its robustness.

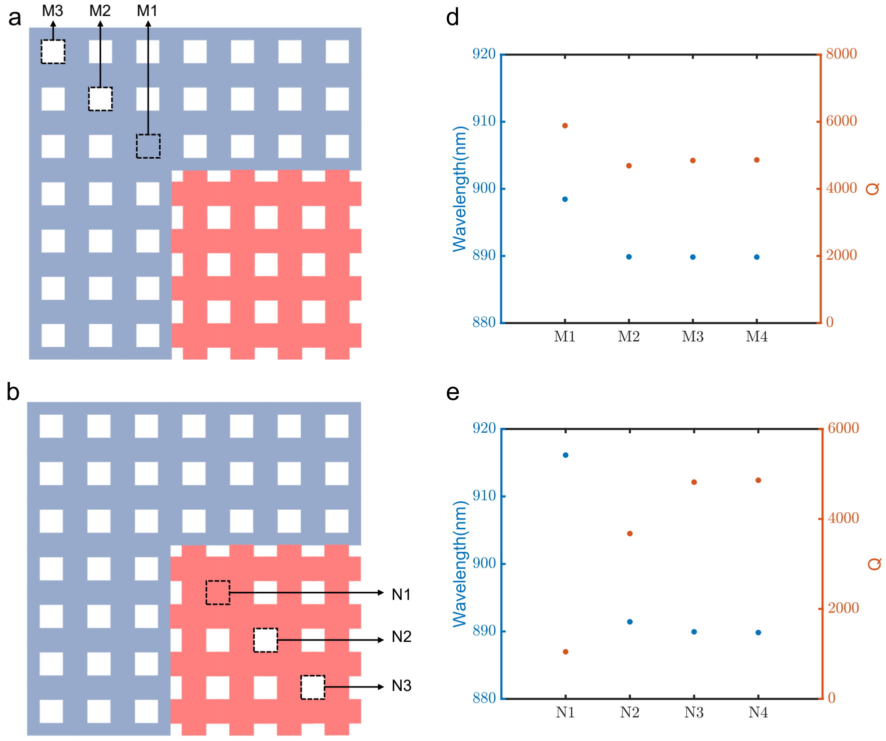

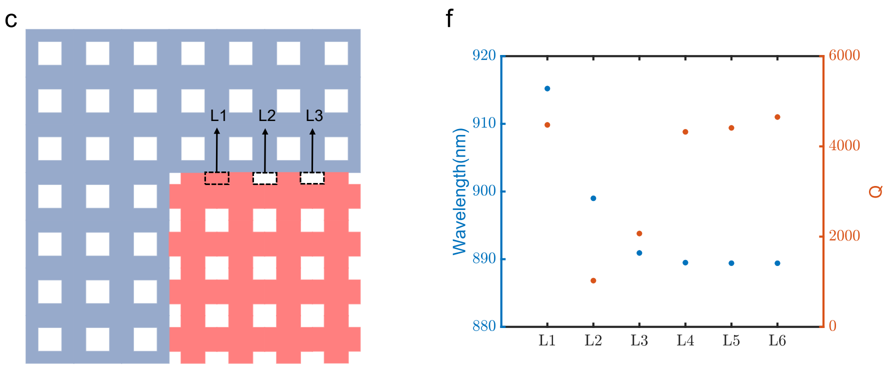

Figure S8 (a-c) Schematic of the edge defect and bulk defect in trivial and nontrivial lattice. The dashed line represents the removing airhole. (d-f) Simulated Q factor and resonance wavelength of the corner state with different defects corresponding to (a-c).

We further discuss bulk defects and edge defects in the sample without the center airhole. The original Q factor of the corner state in the sample without the center airhole is 13192, and the original wavelength is 901.428 nm. Figure S9 illustrates the schematic of forming a defect by removing a hole, using the same procedure as depicted in Figure S8. The effect of all types of defects on wavelength and Q is similar to that observed without the center hole. As the position of the defect moves away from the corner, the mode wavelength gradually blueshifts, and the Q factor rises, approaching the level of the defect-free corner state. This trend holds true for all defects except for the nearest holes N1 and L1. The disappearance of the corner states after removing holes N1 and L1 can be attributed to the removal of the hole at the corner, leading to a reduction in the robustness of the topological protection. After restoring the center hole, as shown in Figure S8 (b-c), we re-observed the existence of the corner state in samples with N1 and L1 defects, proving the robustness of the corner state.

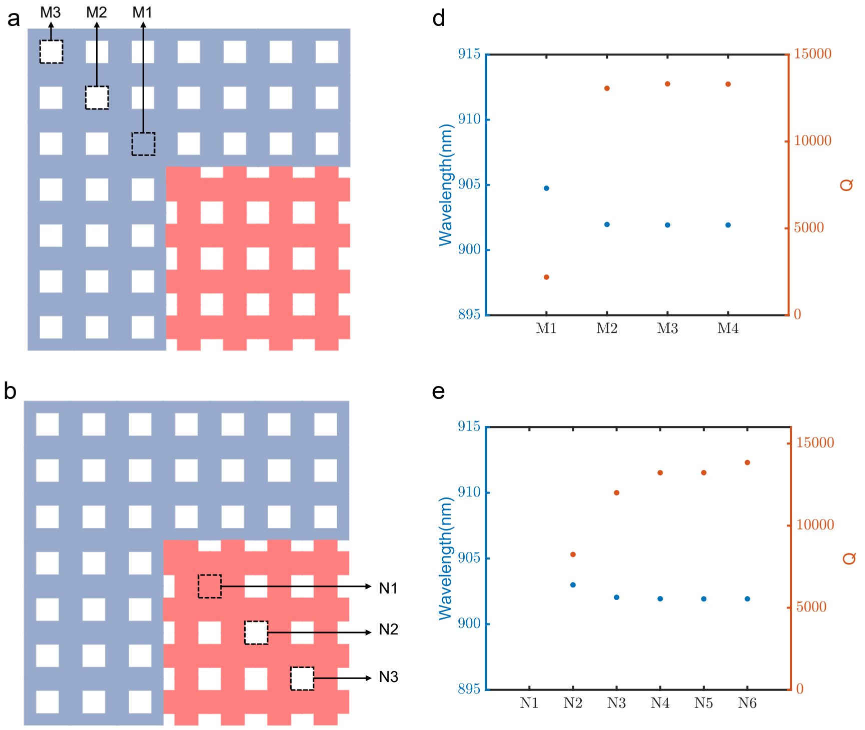

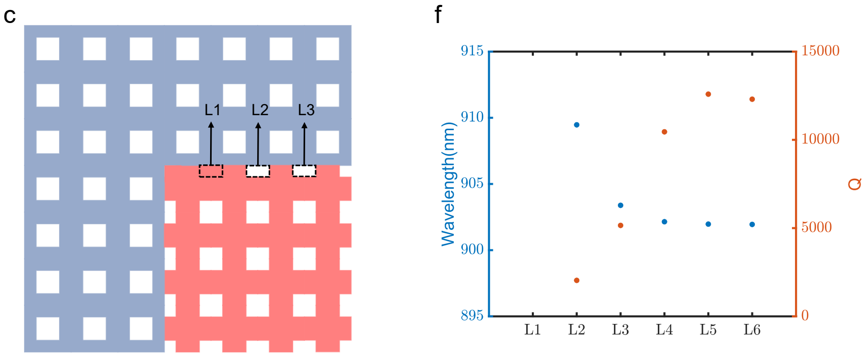

Figure S9 (a-c) Schematic of the edge defect and bulk defect in trivial and nontrivial lattice with hole fulfilled at the corner. The dashed line represents the removing airhole. (d-f) Simulated Q factor and resonance wavelength of the corner state with different defects corresponding to (a-c).

Reference.

1. Johnson, S. & Joannopoulos, J. Block-iterative frequency-domain methods for Maxwell's equations in a planewave basis. *Opt. Express* **8**, 173 (2001).
2. Xie, X. *et al.* Cavity Quantum Electrodynamics with Second-Order Topological Corner State. *Laser & Photonics Reviews* **14**, 1900425 (2020).
3. Xie, X. *et al.* Optimization and robustness of the topological corner state in second-order topological photonic crystals. *Opt. Express* **29**, 30735 (2021).
4. Khitrova, G., Gibbs, H. M., Kira, M., Koch, S. W. & Scherer, A. Vacuum Rabi splitting in semiconductors. *Nature Phys* **2**, 81–90 (2006).
5. Cui, G. & Raymer, M. G. Emission spectra and quantum efficiency of single-photon sources in the cavity-QED strong-coupling regime. *Phys. Rev. A* **73**, 053807 (2006).
6. Englund, D. *et al.* Controlling the Spontaneous Emission Rate of Single Quantum Dots in a Two-Dimensional Photonic Crystal. *Phys. Rev. Lett.* **95**, 013904 (2005).
7. Novotny, L. Strong coupling, energy splitting, and level crossings: A classical perspective. *American Journal of Physics* **78**, 1199–1202 (2010).
